# USING PRE-EXISTING RELATIONSHIPS TO CREATE PIPELINES OF DISASTER PREPAREDNESS SUPPORT FOR HOME HEALTH AGENCIES

**DOI:** 10.1093/geroni/igae098.1606

**Published:** 2024-12-31

**Authors:** Tamar Wyte-Lake, Lauren Hall, Emily Solorzano, Emily Franzosa

**Affiliations:** U.S. Department of Veterans Affairs (VA), North Hills, California, United States; James J Peters VA Medical Center, Bronx, New York, United States; U.S. Department of Veterans Affairs (VA), Los Angeles, California, United States; James J Peters VA Medical Center, Bronx, New York, United States

## Abstract

Home-health agencies (HHAs) provide community-based services that are a vital lifeline for older adults and can play a critical role in protecting older adults’ health and independence during disasters. Yet, HHAs are often under-resourced and disconnected from broader community emergency planning efforts, and therefore can struggle to maintain services during such events, putting clients’ health and safety at risk. Healthcare coalitions have demonstrated a variety of ways to effectively share critical resources during disasters, strategies that could be leveraged to better support community HHAs. Through interviews with 19 stakeholders from six Veterans Health Administration (VA) Medical Centers and associated, contracted HHAs, we used the VA as a case study to explore how interorganizational relationships can be leveraged to maintain continuous care across providers during disasters. VA staff demonstrated interest in partnering around emergency management and identified a range of low- and higher-effort resources, from informational bulletins to staff training and emergency plan preparations that could be shared with HHAs. Creating a pipeline of support through existing relationships can provide a model for health systems to extend available emergency resources and make clinical care teams, patients, and communities more resilient to disasters. We will focus on specific resources and strategies and how to develop and sustain those cross-sector relationships.

